# Low-density lipoprotein decorated silica nanoparticles co-delivering sorafenib and doxorubicin for effective treatment of hepatocellular carcinoma

**DOI:** 10.1080/10717544.2018.1531953

**Published:** 2018-12-27

**Authors:** Junfeng Ye, Ruoyan Zhang, Wengang Chai, Xiaohong Du

**Affiliations:** Department of Hepato-Biliary-Pancreatic Surgery, First Hospital of Jilin University, Changchun, PR China

**Keywords:** Low-density lipoprotein, silica nanoparticles, sorafenib, doxorubicin, hepatocellular carcinoma

## Abstract

Combinational therapy is usually considered as a preferable approach for effective cancer therapy. Especially, combinational chemotherapies targeting different molecular targets are of particular interest due to its high flexibility as well as efficiency. In our study, the surface of silica nanoparticles (SLN) was modified with low-density lipoprotein (LDL) to construct platform (LDL-SLN) capable of specifically targeting low-density lipoprotein receptors (LDLRs) that overexpressing in hepatocellular carcinoma (HCC). In addition, the versatile drug loading capacity of LDL-SLN was employed to fabricate a preferable drug delivery system to co-deliver sorafenib (Sor) and doxorubicin (Dox) for combinational chemotherapy of HCC. Our results revealed that the LDL-SLN/Sor/Dox nanoparticles with size around 100 nm showed preferable stability in physiological environments. Moreover, the LDL-SLN/Sor/Dox could target LDLR overexpressed HepG2 cells. More importantly, both *in vitro* and *in vivo* experiments demonstrated that the LDL-SLN/Sor/Dox exerted elevated antitumor efficacy compared to Sor or Dox alone, which indicated that LDL-SLN/Sor/Dox could be a powerful tool for effective combinational chemotherapy of HCC.

## 1. Introduction

Combinational chemotherapy is generally recognized as a preferable approach for cancer therapy due to its superiority in overcoming drug resistance and heterogeneity of cancer cells (Sui et al., 2017; Li et al., 2018). It is usually achieved by simultaneously delivering different chemotherapeutics with different pharmacological action mechanisms to the same target cell (Liu et al., 2017; Yan et al., 2017). Sorafenib (Sor) is a water-insoluble tyrosine kinase inhibitor that widely employed to inhibit vascular endothelial growth factor and platelet-derived growth factor receptors (Silva et al., 2016; Zhang et al., 2017). Recently, Sor was further approved by FDA to serve as the clinical drug for patients with unresectable hepatocellular carcinoma (HCC) (Xiao et al., 2016). However, clinical observations reveal that Sor displays only cytostatic effects rather than cytotoxicity, which results in the disappointing fact that other compensatory oncogenic pathways were activated to facilitate the cancer cells to evade pharmacotherapy (Thapa et al., 2016; Thomas & Balthasar, 2016). As a result, Sor should be used along with other cytotoxic drugs to achieve better chemotherapy effects.

Doxorubicin (Dox) is a widely adopted antitumor drug with effective DNA intercalation capability, which can significantly disrupt or block the replication processes in a variety of cancers (Wang et al., 2017). However, the undesirable adverse side effects, especially severe cardiotoxicity, of Dox usually hinder its further clinical applications (Allam et al., 2017; Wang et al., 2018; Yang et al., 2018). As a result, Sor and Dox with completely different pharmacokinetic profiles have achieved limited therapeutic success which combined the administration of these two drugs. Nevertheless, low bioavailability as well as poor water solubility, seriously reduce the advantages and restrict the application of combinational chemotherapy.

In recent years, nanoscale drug delivery systems (DDS), especially inorganic ones, have been designed with the purpose to encapsulate both drugs into one vector with desired ratios and specifically deliver the cargos to neoplastic tissues (Baeza et al., 2017; Wu et al., 2017; Tang et al., 2018). Inorganic materials including gold nanoparticles and silica nanoparticles (SLNs), have attracted the interests of many researchers (Rizwan et al., 2017; Ao et al., 2018). The introduction of SLNs for biomedical application is a milestone in cancer therapy, since the versatile capabilities of SLNs, including high biocompatibility and facile surface modification, have made it a superior carrier to other counterparts (Song et al., 2016). Moreover, SLNs also possess decent drug loading capacity toward a variety of drugs which is prerequisite for effective cancer therapy (Lv et al., 2017).

It has been widely acknowledged that the expression of cell surface low-density lipoprotein receptors (LDLR) is usually upregulated in many cancer cell lines, including breast carcinoma, prostate carcinoma, and HCC (Liang et al., 2017). It thus offers an alternative approach to mediate homing of DDS to tumor tissues. Low-density lipoprotein (LDL) as the major cholesterol transporter in the plasma (Xiong et al., 2017), has been approved to have high affinity to LDLR according to a previous report (Su et al., 2016). As a result, the endogenic LDL with high biocompatibility and low cytotoxicity is widely considered to be suitable for the development of ideal DDSs (Zhu et al., 2017; Yesylevskyy et al., 2018), which has been practiced by various previous reports (Zhang et al., 2016; Gong et al., 2017).

We herein developed LDL modified lipidic silica nanoparticles (LDL-SLN) as a preferable DDS for the co-delivery of Sor and Dox, with the aim to construct a DDS (LDL-SLN/Sor/Dox) that able to deliver two drugs (Sor and Dox) specifically to the tumor site via LDLR mediated tumor-homing property, which was expected to achieve enhanced antitumor effect compared to unmodified SLNs and single drug.

## 2. Materials and method

### 2.1. Materials

Triton X-100, *N*-(2-aminoethyl)-3-aminopropyltrimethoxysilane and tetraethyl orthosilicate were purchased from Aladdin (Shanghai, China). Plasma-derived LDL was obtained from Intracel (Frederick, MD, Germany). Sor, DOX, 3-(4,5-dimethylthiazol-2-yl)-2,5-diphenyl tetrazolium bromide (MTT), and DiR were obtained from Sigma (St. Louis, MO, USA).

### 2.2. Cell culture and animal model

Human liver cancer cell line (HepG2) from American Type Culture Collection (ATCC, USA) was maintained in DMEM (Gibco, USA) containing 10% (*v*/*v*) fetal bovine serum (Gibco) and penicillin/streptomycin (Gibco, 100 U/mL) in an incubator at 37 °C (MCO-18AIC, SANYO, USA) with humid atmosphere (5% CO_2_/95% air).

New Zealand rabbit as well as female Balb/c nude mice (∼22 g) were purchased from Shanghai SLAC Laboratory Animal Co., Ltd. (Shanghai, China). The animals were housed in homothermal (25 ± 2 °C) SPF-II house (25 ± 2 °C) with full access to diet. The HepG2 tumor xenograft model was established according to the previous report by subcutaneously injection HepG2 cells (1 × 10^7^ cells/mL in saline) into the flank of mouse and then allow to grow for 2–3 weeks into solid tumors (Zhao et al., 2018). All procedures were conducted in line with NIH guidelines and approved by the Ethics Committee of First Hospital of Jilin University.

### 2.3. Preparation of LDL-SLN/Sor/Dox

Amine-modified SLN was firstly synthesized in a water-in-oil microemulsion with minor modification as previously reported (Wu et al., 2015). Later, the as prepared amine-modified SLN was subjected to drug loading (Ao et al., 2018). In brief, SLN was resuspended in mixed solution (ethanol:pyridine =1:1, *v*/*v*) and incubated with Sor and Dox (5 mg/mL) for 30 min. Subsequently, the drug-loaded SLN (SLN/Sor/Dox) was isolated using high-speed centrifugation (8125 rpm, 10 min, CR21, Hitachi, Japan). The drug loading content (DLC) was calculated by determining the remaining Sor and Dox in the supernatant using UV-spectrophotometer (DR6000, HACH, USA). The concentrations of Sor and Dox in the supernatant were diluted in acidic DMSO and measured by a UV-spectrophotometer. The calibration curve for the Sor and Dox content calculations was obtained by measuring the absorbance of Sor and Dox solutions of various concentrations in DMSO at 282 and 490 nm for Sor and Dox, respectively.

To finally construct the LDL-SLN/Sor/Dox, SLN/Sor/Dox was resuspended in an aqueous solution containing LDL (1 mg/mL) under gentle agitation at room temperature. After 6 h of incubation, the LDL-SLN/Sor/Dox was obtained using high-speed centrifugation.

### 2.4. Measurement of particle size and zeta potential

Samples were assessed at 25 °C using a Zeta plus zeta potential analyzer (Morphologi G3-ID, Malvern, UK). The morphology of nanoparticle was observed by scanning electron microscope (SEM, GeminiSEM, Zeiss, Germany).

### 2.5. Colloidal stability and hemolysis assays

The colloidal stability and hemolysis assays for LDL-SLN/Sor/Dox were performed according to the previous reports (Tang et al., 2018). In brief, the change in particle size of LDL-SLN/Sor/Dox diluted with phosphate buffer (PBS, pH 7.4) was recorded for up to 48 h. For hemolysis assay, LDL-SLN/Sor/Dox was added into 2% red blood cells (RBCs) suspension to achieve the designated concentrations and incubated at 37 °C for 1 h. After being centrifuged at 3000 rpm for 10 min, absorption values of the supernatants were measured by UV spectrophotometer.

### 2.6. *In vitro* drug release

The release profile of Sor and Dox from LDL-SLN/Sor/Dox was explored using dialysis method according to the previous report (Meng et al., 2018). In brief, LDL-SLN/Sor/Dox was suspended in PBS (0.1% Tween 80, *w*/*v*) with different pH (7.4 and 5.0). At predetermined intervals, the aliquot solution was withdrawn and the drug content within was determined.

### 2.7. Cellular uptake of LDL-SLN/Sor/Dox

The time-dependent intracellular uptake profile of LDL-SLN/Sor/Dox in HepG2 cells was quantitatively assessed using flow cytometer (FCM, BD FACSCalibur™, USA). HepG2 cells were seeded and cultured in 24-well plates to reach 90% confluence. After incubation with fresh culture medium containing SLN/Sor/Dox or LDL-SLN/Sor/Dox for different time intervals, the cells were detached and subjected to measure the intracellular Dox fluorescence intensity. A minimum of 1 × 10^4^ cells were randomly selected from each sample and the mean fluorescence intensity of these cells was calculated. Moreover, cells incubated with excess LDL (150 μg/mL, 1 h) prior to sample addition was also conducted to investigate if the internalization of LDL-SLN/Sor/Dox was related to LDL.

### 2.8. Cytotoxicity activity

The cytotoxicity of LDL-SLN/Sor/Dox was also studied on HepG2 cells. In detail, HepG2 cells were seeded and cultured in 96-well plates to reach 60% confluence. Later, the primary medium was discarded and cells were further incubated with fresh ones containing different samples. Sor and Dox concentration were set at 3.5 μg/mL and 0.5 μg/mL, respectively. After 48 h of incubation, a standard MTT assay was conducted as previously reported (Wang et al., 2015).

### 2.9. Apoptosis assay

Cells were harvested and the fraction of apoptotic cells determined by Annexin V apoptosis kit (Invitrogen, Carlsbad, CA) for flow cytometry analysis following the manufacturer’s instructions.

Total protein was extracted from the tissues and cell lines using the RIPA buffer consisting of 50 mM Tris pH 7.4, 150 mM NaCl, 1% NP-40, 0.5% sodium deoxycholic acid and 0.1% sodium dodecyl sulfate as reported 27. The protein sample was quantified using the Bradford Protein Concentration Determination Kit (Beyotime, Haimen, Jiangsu, China). The same amount of protein sample was separated using a 10% SDS-PAGE gel and transferred to a polyvinylidene difluoride membrane (EMD Millipore Corporation, Billerica, MA) at 80 V for 2 h at 4 °C. The membrane was blocked with fat-free milk for 1 h at 4 °C, and subsequently incubated with the primary antibodies (Abcam, Cambridge, MA). On the next day, the membrane was washed with TBS-T for three times and incubated with a horseradish peroxidase-conjugated secondary antibody (Abcam, Cambridge, MA) for 2 h at room temperature. Protein bands were visualized using a BeyoECL Plus Kit (Beyotime, Haimen, Jiangsu, China) and quantified using the Tanon Automatic Chemiluminescence Western Blot Imaging system (Tanon, Shanghai, China).

### 2.10. *In vivo* tumor-targeting of LDL-SLN/Sor/Dox

DiR as a near-infrared fluorescent probe was encapsulated during the drug loading process. The HepG2 xenograft model established in Section 2.2 was intravenously injected with DiR loaded LDL-SLN/Sor/Dox and SLN/Sor/Dox (DiR dosage: 10 μg/mouse). At 24 h post injection, the mice were sacrificed and their tumor tissues as well as major organs were harvested. The *in vivo* tumor targeting efficacy of different nanoparticles was evaluated using the *In Vivo* Imaging System (QuickView3000, Bio-Real, Austria).

### 2.11. *In vivo* antitumor efficacy

The *in vivo* antitumor efficacy of LDL-SLN/Sor/Dox was investigated using HepG2 tumor xenograft models. The mice were adopted for *in vivo* experiments when the tumor volume over 100 mm^3^. All mice were divided into five groups (*n* = 6) randomly: (1) saline (control); (2) free Sor; (3) free Dox; (4) SLN/Sor/Dox; (5) LDL-SLN/Sor/Dox. The nanoparticles were intravenously administrated (35 mg/kg Sor and/or 5 mg/kg Dox per mouse) for 7 times over 14 days (Lin et al., 2016). The body weights as well as tumor sizes were recorded before injection.

## 3. Results and discussions

### 3.1. Particle size, dispersity, morphology, and drug loading of LDL-SLN/Sor/Dox

**Figure 1.**
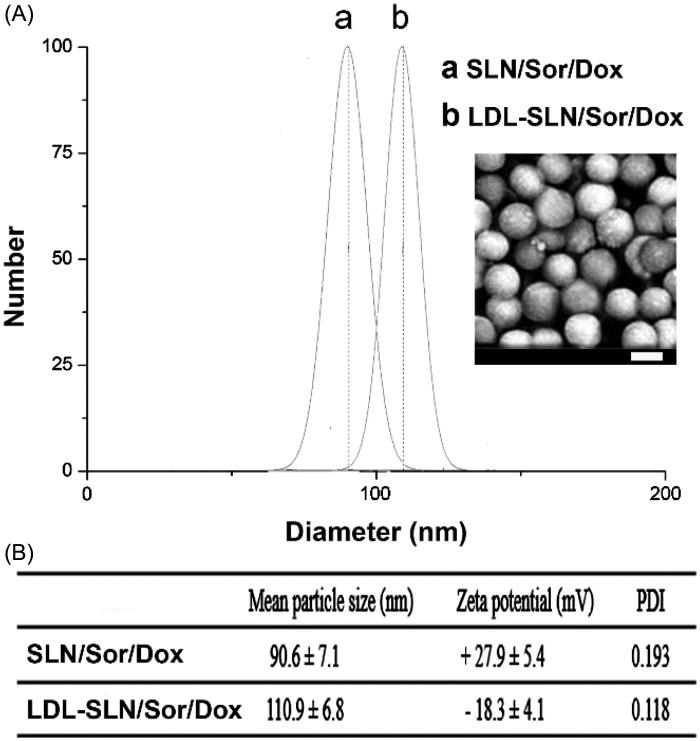
Particle size distribution (A) of SLN/Sor/Dox and LDL-SLN/Sor/Dox. Inserted image is the morphology of LDL-SLN/Sor/Dox obtained by SEM (B) Mean particle size, zeta potential, and poly dispersion index (PDI) measurements of amine decorated SLNs and LDL/SLNs. Data were shown as mean ± S.D. (*n* = 3).

Amine decorated SLNs have widely adopted as a powerful tool to construct multifunctional DDSs due to the existence of excess primary amine groups on the surface that further modification of other functional groups could be easily achieved. The particle size of dual drug loaded SLN/Sor/Dox was shown in Figure 1(A). The results revealed that SLN/Sor/Dox was composed of nano-sized particles with a diameter of around 90.6 nm and a relatively small PDI of 0.193. It was suggested that the LDL was anchored on the surface of SLN/Sor/Dox through electrostatic adsorption. As displayed in Figure 1(A), compared to that of SLN/Sor/Dox, the resulted LDL-SLN/Sor/Dox showed an increase in particle size to around 110.9 nm with a decreased PDI of 0.118. The decreased PDI value of LDL-SLN/Sor/Dox, in line with previous reports (Stoffelen et al., 2015; Ruff et al., 2016), suggested that modification with hydrophilic component could be beneficial to the dispersion of nanoparticles. In addition, zeta potential measurement was performed to further prove the successful modification of LDL. As shown in Figure 1(B), SLN/Sor/Dox was positively charged particles with a surface charge of +27.9 mV. In contrast, the surface potential of LDL-SLN/Sor/Dox was reversed after modification of LDL to be −18.3 mV which beneficial for LDL-SLN/Sor/Dox to bypass the complicated extracellular barriers within the circulation system to ensure effective delivery of encapsulated drugs (Abbad et al., 2015).

The large surface area and pore volume of SLNs are able to achieve decent drug loading of a variety of molecules. In our study, by carefully tuning the ratio of Sor and Dox and controlling the drug loading process, we successfully co-loaded Sor and Dox in one carrier with a *w*/*w* ratio of 7 (The DLC for Sor and Dox was 37.1% and 5.3%) according to our HPLC analysis, which is sufficient for the following *in vitro* and *in vivo* assays.

### 3.2. *In vitro* drug release

**Figure 2.**
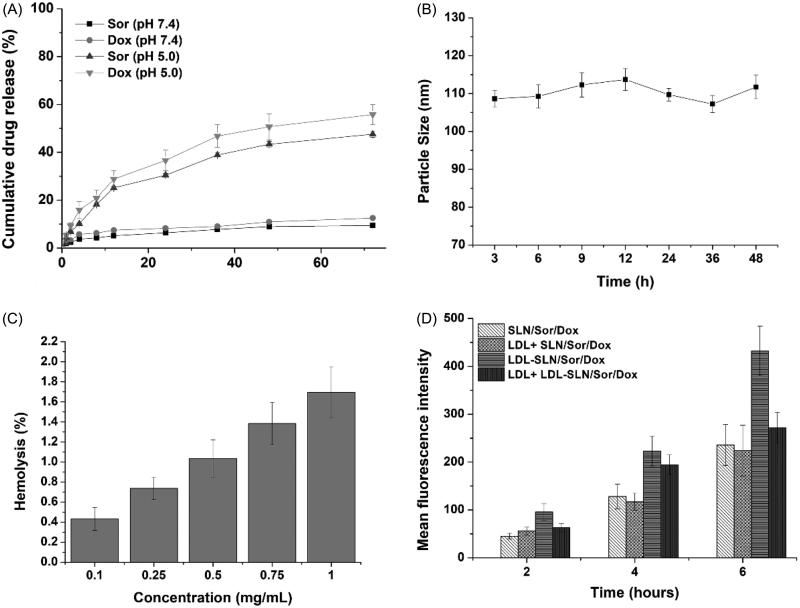
(A) *In vitro* drugs (Sor and Dox) release of LDL/SLN/Sor/Dox under different pH 7.4 and 5.0. (B) Colloidal stability of LDL/SLN/Sor/Dox in PBS (37 °C, 48 h). (C) Hemolysis of LDL/SLN/Sor/Dox at various concentrations. (D) *In vitro* time-dependent (2, 4, and 6 h) quantitative FCM analysis SLN/Sor/Dox and LDL/SLN/Sor/Dox with/without LDL pretreatment on HepG2 cells. Data were expressed as mean ± S.D. (*n* = 3).

With the aim to investigate the drug release profile of LDL-SLN/Sor/Dox in different conditions, PBS with different pH values were adopted. The pH 7.4 simulates the extracellular physiological environment and pH 5.0 mimicking the intracellular environment of cancer cells, since all of the cancer cells have a relatively acidic pH conditions when compared with normal cells or blood conditions. As displayed in Figure 2(A), drug release of both Sor and Dox was less than 15% in 72 in pH 7.4, which indicated that the drug leakage of LDL-SLN/Sor/Dox was relatively slow under physiological condition. This is beneficial for LDL-SLN/Sor/Dox to avoid potential side effects for safe and effective drug delivery to the neoplastic cells (Bian et al., 2015; Shi et al., 2016). In contrast, the drug release of both Sor and Dox under the acidic condition (pH 5.0) was significantly accelerated. In detail, 47.6% of the encapsulated drug was released (72 h), which was three time of that of in pH 7.4 under the same condition. This phenomenon was also observed in Dox release (55.8%). The accelerated drug release in acidic environment guarantees the rapid transformation of LDL-SLN/Sor/Dox into available Sor and Dox within tumor cells, which is beneficial for the distinguished drug release in neoplastic cells instead of in normal tissues to avoid the potential side effects of chemotherapy and to increase its antitumor efficacy.

### 3.3. Stability test

It has been generally recognized that several basic requirements are needed if a DDS is intended to safely deliver the encapsulated cargos. Since the particle size plays a critical role in determining the *in vivo* fate of the DDS, with the aim to bypass multiple extracellular barriers, the DDS should be capable of maintaining its morphology without significant size change long enough before arriving at the targeted sites (Hashemi et al., 2016). As a result, LDL-SLN/Sor/Dox was studied regarding its stability as a function of time. To assess the colloidal stability of LDL-SLN/Sor/Dox under physiological conditions, the change in particle size in PBS was monitored for 48 h. As shown in Figure 2(B), the size of LDL-SLN/Sor/Dox remained stable during without significant change during the whole time. It was therefore suggested that LDL-SLN/Sor/Dox was able to maintain its size under physiological environment for a relatively long time.

Hemolysis as an indicator for potential risks in medical application was also investigated. Results obtained from Figure 2(C) showed that LDL-SLN/Sor/Dox exerted neglectable hemolysis (1.65%) on RBCs even at the highest concentration (1 mg/mL), which was much higher than the actual *in vivo* blood concentration (due to dilution and distribution). As a result, it was concluded that LDL-SLN/Sor/Dox could be a safe nanoparticle with low hemolysis potential.

### 3.4. Cellular uptake of LDL-SLN/Sor/Dox

It has been reported by previous researches that LDL modification on the surface of the DDS can improve its targeting to LDLR, a receptor excessively expresses on various types of cancer, including hepatocellular cancer (Tol et al., 2010; Xu et al., 2016; Wang et al., 2017). As a proof of concept, Dox was employed as the indicator to report the cellular uptake profile different samples by FCM at different time points.

As illustrated in Figure 2(D), the gradually increased fluorescence signal in cells as a function of time suggested that the cellular uptake of both nanoparticles was positively related to incubation time. Additionally, it was noted that higher Dox fluorescence signals were observed in LDL-SLN/Sor/Dox group at all tested time intervals, which was 1.83-fold of that in SLN/Sor/Dox group after 6 h of incubation, which suggested that LDL-SLN/Sor/Dox can be readily internalized into HepG2 cells. In order to verify whether internalization of LDL-SLN/Sor/Dox was via the LDLR mediated endocytosis, cells were pretreated with LDL for 2 h prior to formulation. It was interesting to observe that the fluorescence intensity of LDL-SLN/Sor/Dox group suffered a great decline at all time intervals while that in SLN/Sor/Dox group stayed at almost the same level. It was clearly demonstrated by these results that LDL-SLN/Sor/Dox was internalized into cells through LDLR-related endocytosis.

### 3.5. Cytotoxicity assay

**Figure 3.**
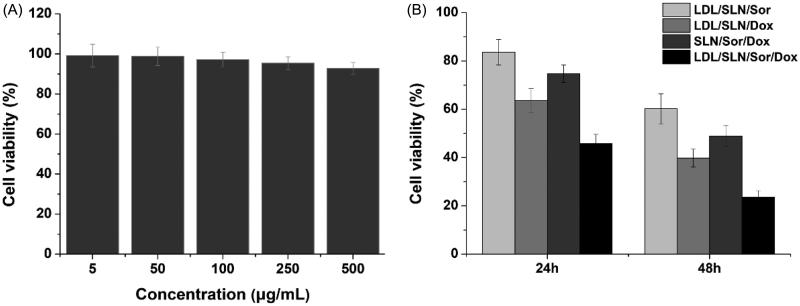
(A) Cytotoxicity of free LDL/SLNs on HepG2 cells at 48 h post incubation. (B) Cytotoxicity of free Sor, free Dox, SLN/Sor/Dox and LDL/SLN/Sor/Dox against HepG2 cells after 24 and 48 h of incubation. Sor and Dox concentration was set at 3.5 μg/mL and 0.5 μg/mL, respectively. Data were expressed as mean ± S.D. (*n* = 3).

The *in vitro* synergetic effect of Sor and Dox using the well-designed LDL-SLN/Sor/Dox was further investigated using MTT assay. The cytotoxicity of drug-free LDL/SLNs was firstly studied (5–500 µg/mL) to exclude potential false positive results caused by the carrier. As displayed in Figure 3(A), no significant toxic (cell viability over 90%) effect was observed in all adopted concentrations. It thus concluded that the LDL/SLNs was biocompatible to grant a broad range in both cancer therapy and other biomedical fields. Later, HepG2 cells were treated with LDL-SLN/Sor/Dox under different drug concentrations (Figure 3(B)). It was interesting to note that administration of Sor and Dox (LDL/SLN/Sor and LDL/SLN/Dox) alone have only moderate antitumor effects on HepG2 cells and this effect was positively related to incubation time as cells after 48 h of incubation showed much lower cell viability as compared to that of 24 h. Moreover, it was interesting to find out that the co-delivery of Sor and Dox (LDL/SLN/Sor/Dox) exerted much more elevated antitumor effect than either mono formulations at all the adopted drug concentrations, which indicated that the Sor and Dox with synergetic effect might exert much effective chemotherapy outcome than applying anyone of them alone. In addition, as confirmed by cellular uptake experiment that LDL modification can increase the intracellular drug level, the viability of cells treated with LDL/SLN/Sor/Dox is much lower than SLN/Sor/Dox under the same conditions, which was in line with previous report ( Ao et al., 2018).

### 3.6. Apoptosis assays

**Figure 4.**
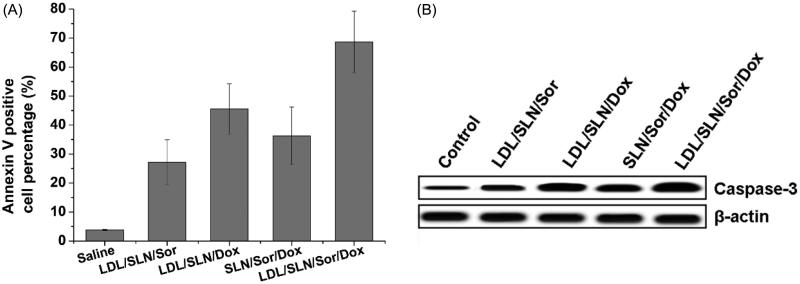
Apoptosis assay of LDL/SLN/Sor/Dox. (A) Annexin V staining and (B) western blotting assay of HepG2 cells treated with different formulations. Data were shown as mean ± S.D. (*n* = 3).

It is well established that HepG2 cells with Sor and Dox result in the decrease of survival as revealed by cytotoxicity assay. However, whether cell death induction is also involved in reducing viability remains to be unknown. We therefore attempted to delineate if apoptosis induction was involved in decreasing viability in the HepG2 cells following Sor and Dox treatment by interrogating established cell death markers such as annexin V staining and Caspase 3. As depicted in Figure 4(A), the percentage of annexin V cells positive cells was greater in Sor treated group compared to the control (untreated). The fraction of annexin V positive cells increased from approximately 3.8% in control to 12.3% in the Sor treated samples (*p* < .01), indicating that apoptosis was involved in Sor treatment. In line with previous reports, Dox treatment also increased fraction of annexin V positive cells to. It was also noted that in consistence with cytotoxicity assay, co-delivery of Sor and Dox achieved higher apoptosis percentage than treated with Sor or Dox alone. On the other hand, LDL-SLN/Sor/Dox with LDL modification showed higher cell deaths percentage to SLN/Sor/Dox, which indicated that LDL modification was capable of increasing the apoptosis effects of the DDS. Western blot analysis was also conducted (Figure 4(B)) and further confirmed this conclusion.

### 3.7. *In vivo* imaging of LDL-SLN/Sor/Dox

**Figure 5.**
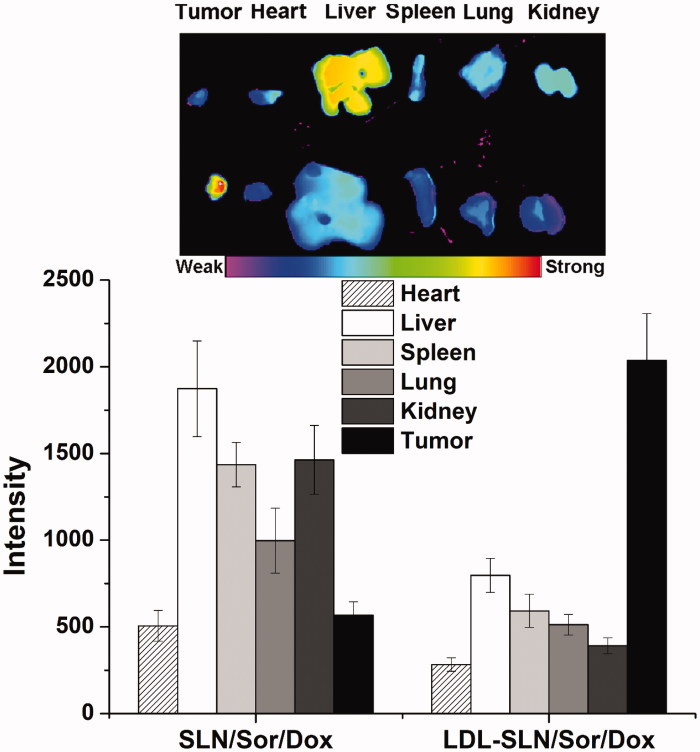
Quantitative *ex vivo* mean fluorescence intensity of dissected major organs and tumor at 24 h post injection of DiR labeled SLN/Sor/Dox (upper) and LDL/SLN/Sor/Dox (lower). Data were expressed as mean ± S.D. (*n* = 3).

LDL modification was supposed to increase the homing of LDL-SLN/Sor/Dox to the tumor site. In order to prove this conjecture, the distribution of DiR in major organs as well as tumor tissue after 24 h of injection was observed using an NIR fluorescence imaging system. Figure 5 showed the results obtained from *ex vivo* imaging. It was concluded that due to its poor tumor targeting capability, SLN/Sor/Dox was mainly accumulated in the liver and kidney. On the contrary, LDL modification could help the nanoparticles escape the liver capture to significantly increase their accumulation to the tumor. In summary, LDL-SLN/Sor/Dox demonstrated more preferable tumor homing property than SLN/Sor/Dox, which might due to LDLR-mediated targeting mechanism.

### 3.8. *In vivo* antitumor efficacy

**Figure 6.**
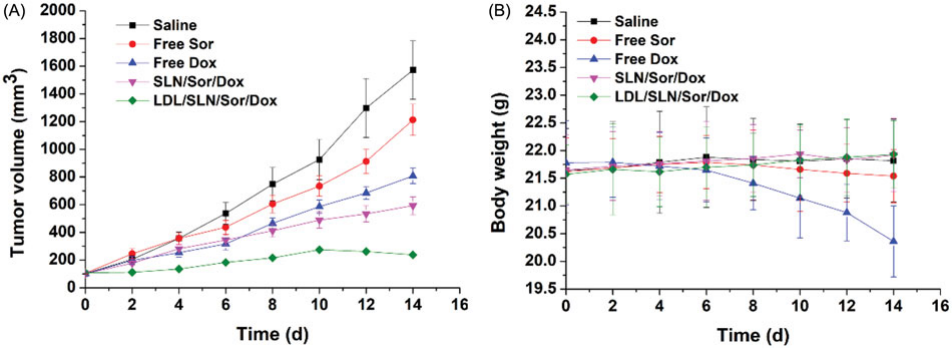
The tumor volume (A) and body weight (B) analysis of HepG2 tumor-bearing model after intravenous injection administration of saline, free Sor, free Dox, SLN/Sor/Dox and LDL/SLN/Sor/Dox, respectively (35 mg/kg Sor and/or 5 mg/kg Dox each mouse). Data were expressed as mean ± S.D. (*n* = 6).

The *in vivo* antitumor capability of LDL-SLN/Sor/Dox in established HepG2 xenografted model. LDL-SLN/Sor/Dox, as well as SLN/Sor/Dox and free drugs (Sor and Dox) were assessed relating to their capability to suppress tumor growth as compared with saline. As displayed in Figure 6(A), it was interesting to find that both the antitumor effect of LDL-SLN/Sor/Dox and SLN/Sor/Dox were superior to free drugs, which was opposite to the results obtained in cytotoxicity assay. It was inferred that due to the enhanced tumor homing property, DDS was able to deliver more drugs to the tumor tissue compared with the free drug (Xiong et al., 2018). In addition, it was noted that LDL-SLN/Sor/Dox exerted the most potent antitumor efficacy compared with other groups with the smallest tumor volumes of 181 ± 16 mm^3^. Moreover, body weight variations as a function of the time and formulation were recorded. As shown in Figure 6(B), free drugs, especially Dox showed strong system toxicity with a steady loss in body weight (since day 4) observed during the whole experiment. In addition, Sor treated mice began to lose their body weight at day 10, suggesting that the health condition of mice was compromised due to the side effects of free drugs. However, no noticeable loss in body weight was observed in LDL-SLN/Sor/Dox-treated group.

## 4. Conclusion

In our study, LDL modified SLNs was developed to co-deliver Sor and Dox (LDL-SLN/Sor/Dox). It combines the tumor homing property of LDL as well as the drug loading ability of SLNs for effective synergetic chemotherapy of hepatocellular cancer. Our experimental results revealed that LDL-SLN/Sor/Dox was nano-sized particles with decent drug loading, which could preserve unwanted drug leakage under physiological condition while accelerated drug release under acidic neoplastic environment. Nevertheless, LDL-SLN/Sor/Dox can increase the intracellular drug concentration in HepG2 cells compared with unmodified ones via the LDLR-mediated endocytosis. It was worth mentioning that LDL-SLN/Sor/Dox exhibited preferable antitumor activity with minimized toxic side effects both *in vitro* and *in vivo* due to the synergetic effect of Sor and Dox.
